# Application of Piezo-Based Measuring System for Evaluation of Nucleic Acid-Based Drugs Influencing the Coagulation

**DOI:** 10.3390/s20010152

**Published:** 2019-12-25

**Authors:** Silju-John Kunnakattu, Ludmilla Hann, Julia Kurz, Hanna Haag, Stefan Fennrich, Nicole Rauch, Christian Schlensak, Hans-Peter Wendel, Sandra Stoppelkamp, Meltem Avci-Adali

**Affiliations:** 1 Department of Thoracic and Cardiovascular Surgery, University Hospital Tuebingen, 72076 Tuebingen, Germany; Silju.kunnakattu@klinikum.uni-tuebingen.de (S.-J.K.); Ludmilla.hann@klinikum.uni-tuebingen.de (L.H.); Julia.kurz@klinikum.uni-tuebingen.de (J.K.); Hanna.haag@klinikum.uni-tuebingen.de (H.H.); stefan.fennrich@gmail.com (S.F.); Christian.schlensak@med.uni-tuebingen.de (C.S.); Hans-peter.wendel@med.uni-tuebingen.de (H.-P.W.); Sandra.stoppelkamp@klinikum.uni-tuebingen.de (S.S.); 2 Department of Micro- and Nanoanalytics, University of Applied Sciences Iserlohn, 58644 Iserlohn, Germany; Rauch.nicole@fh-swf.de

**Keywords:** rheometry, blood coagulation, hemorheology, viscoelastic, piezo, point-of-care, PIEZ

## Abstract

During open-heart surgery, the status of hemostasis has to be constantly monitored to quickly and reliably detect bleeding or coagulation disorders. In this study, a novel optimized piezo-based measuring system (PIEZ) for rheological monitoring of hemostasis was established. The applicability of the PIEZ for the evaluation of nucleic acid-based drugs influencing coagulation was analyzed. Thrombin aptamers such as NU172 might be used during extracorporeal circulation (ECC) in combination with a reduced heparin concentration or for patients with heparin-induced thrombocytopenia (HIT). Therefore, the effect of the coagulation inhibiting thrombin aptamer NU172 and the abrogation by its complementary antidote sequence (AD) were investigated by this rheological PIEZ system. After the addition of different NU172 concentrations, the coagulation of fresh human blood was analyzed under static conditions and using an in vitro rotation model under dynamic conditions (simulating ECC). The clotting times (CTs) detected by PIEZ were compared to those obtained with a medical reference device, a ball coagulometer. Additionally, after the circulation of blood samples for 30 min at 37 °C, blood cell numbers, thrombin markers (thrombin-antithrombin III (TAT) and fibrinopeptide A (FPA)) and a platelet activation marker (β-thromboglobulin (β-TG)) were analyzed by enzyme-linked immunosorbent assays (ELISAs). The increase of NU172 concentration resulted in prolonged CTs, which were comparable between the reference ball coagulometer and the PIEZ, demonstrating the reliability of the new measuring system. Moreover, by looking at the slope of the linear regression of the viscous and elastic components, PIEZ also could provide information on the kinetics of the coagulation reaction. The shear viscosity at the end of the measurements (after 300 s) was indicative of clot firmness. Furthermore, the PIEZ was able to detect the abrogation of coagulation inhibition after the equimolar addition of NU172 aptamer´s AD. The obtained results showed that the established PIEZ is capable to dynamically measure the hemostasis status in whole blood and can be applied to analyze nucleic acid-based drugs influencing the coagulation.

## 1. Introduction

Worldwide, about one million heart surgeries are performed annually, which require extracorporeal circulation (ECC) using the heart-lung machine [1]. The ECC includes various methods, such as dialysis, extracorporeal membrane oxygenation (ECMO) for respiratory support, extracorporeal life support (ECLS), and ventricular assist devices (VAD) for cardiac support [2,3,4]. During ECC, the contact of blood with foreign surfaces can lead to life-threatening activation of coagulation. Therefore, anticoagulants, such as heparin, are used to prevent coagulation activation. Heparin binds to the enzyme inhibitor antithrombin III (AT) and leads to its activation due to conformational change. The activated AT interacts with the exosite II of thrombin and results in the inactivation of thrombin [5]. However, some patients develop hypersensitivity reactions against heparin, such as ‘heparin-induced thrombocytopenia’ (HIT), which leads to a decrease in the platelet counts (thrombocytopenia) [6]. Thus, research is performed to develop new drugs as anticoagulants. The DNA aptamer NU172 is selected against thrombin and specifically binds to the anionic binding site exosite I of thrombin, which has two exosites, exosite I and II. As a result, fibrinogen cannot be transformed into fibrin and the coagulation process is inhibited [5,7]. Thus, coagulation inhibiting aptamers, such as thrombin binding aptamer NU172, represent a promising alternative to the use of heparin in HIT patients during ECC.

Aptamers can fold into 3D structures and bind their targets with a high affinity and specificity [8]. They can be used as theranostics and the modifications at defined positions enable the fine-tuning of their stability and bioavailability [9]. A further great advantage of aptamers is their antagonizability by the use of complementary oligonucleotides, so-called antidotes (ADs). The drug-induced side effects can be significantly reduced by the use of antagonizable drugs and the blocking of their therapeutic effect after treatment. The three-dimensional (3D) structure of the aptamer can be changed by the addition of an AD, which hybridizes to the aptamer and abrogates the binding of the aptamer to its target [10,11,12]. Thereby, the effect of aptamer-based drugs can be abolished. 

ECC can last several hours, thus the continuous monitoring of the coagulation status during and after the ECC is important to prevent bleeding or thrombosis-related complications [13]. Thrombin converts fibrinogen into insoluble fibrin [14], which is then cross-linked with activated platelets, resulting in the generation of a platelet-fibrin network. This leads to an increase in viscous and elastic components of the blood viscosity [15]. Thus, the viscoelastic properties of blood can be detected to monitor the coagulation status in real-time [16]. 

Pechold and co-workers developed a piezo-based device, called piezoelectric axial vibrator (PAV) [17,18]. Using this device, the linear viscoelasticity of soft material fluids, such as blood, and low viscous polymeric solutions can be characterized at higher frequencies (1 to 1000 Hz) compared to currently used rheometers. PAV is able to measure blood coagulation in a physical reliable range with low measuring gaps such as 10 µm, where only a monolayer of blood cells can be formed. 

Currently, modern intensive care does not provide a comprehensive point-of-care (POC) solution for the detection of coagulation status and blood clotting problems. So far, a single medical device, which allows fast and reliable detection of the hemostasis status (coagulation, fibrinolysis, and platelet function) is lacking. The promising results of our recent studies with PAV [19] and oscillatory rheometer [20] indicate that the above mentioned clinical challenges of hemostasis monitoring could be solved by rheology. 

Since the oscillation rheometer is too large and too expensive for the clinical routine and additionally susceptible to mechanical shock, in this study, an optimized piezo-based system (PIEZ) was developed based on PAV. By doubling the diameter of the measuring chamber and reducing the stiffness of the whole system, the optimized PIEZ is able to measure multiple layers of blood cells with a 50 µm gap. Furthermore, this gap height provides more space for the formation of a 3D clot. In contrast to Kirschenmann’s work [17,21], in this study, piezo tubes were used instead of glued piezos on a copper square tube and a heater was integrated. Using the new system, the viscoelastic properties of blood can be determined rheometrically and used to monitor hemostasis. 

In this study, we examined the reliability and applicability of the PIEZ system for the evaluation of nucleic acid-based drugs influencing the coagulation, such as the thrombin aptamer (NU172). Therefore, the coagulation of blood samples was tested under static and dynamic conditions after the addition of NU172 by using PIEZ and a medical reference device (ball coagulometer (KC1A)) in comparison. Furthermore, the inhibition of thrombin aptamer by its AD was analyzed. In addition to the clotting time (CT), further rheological parameters such as the linear slopes (m) of the viscous and elastic components and the viscosity at a certain predefined time point were determined in order to obtain additional information about the coagulation process and clot firmness. During the coagulation, shear viscosity increases over time, and the linear slopes (m) of viscous and elastic components describe the increase of clot formation. The clot firmness, which is dependent on blood fibrinogen level, fibrin cross-linking, and platelet numbers can be obtained by a detected viscosity value at a certain time point. Finally, an in vitro rotation model was used to validate rheologically determined coagulation status. Blood counts were measured, and the thrombin markers (thrombin-antithrombin III (TAT) complex and fibrinopeptide A (FPA)) and the platelet activation marker (β-thromboglobulin (β-TG)) were quantified by enzyme-linked immunosorbent assays (ELISAs) after the incubation of blood with diverse aptamer-combinations. 

## 2. Materials and Methods

### 2.1. Ethics Statement

The Ethics Committee of the University of Tuebingen approved the blood sampling procedures, and all subjects gave written informed consent (project approval number: 270/2010BO1).

### 2.2. Oligonucleotides

The incubation of blood was performed with the thrombin binding aptamer (NU172) and its complementary single-stranded (ss) DNA (AD) (Table 1). Furthermore, a nonsense aptamer (NS) and a nonsense antidote (NS_AD) were used as negative controls [7]. All oligonucleotides were ordered HPLC-purified from Ella Biotech GmbH (Martinsried, Germany).

### 2.3. Blood Collection

Human whole blood was drawn by venipuncture into 3 mL blood collection tubes containing 1 U/mL sodium-heparin (Ratiopharm Merckle GmbH, Germany) or 0.106 mol/L trisodium citrate (S Monovette 3 mL 9NC, Sarstedt AG, Nümbrecht, Germany), which prevents the coagulation by reversible binding to calcium ions.

For all donors (age: 25 to 45 years, male or female), the following exclusion criteria were imperative: Smoking, pregnancy, and taking drugs (particularly drugs affecting the hemostasis, such as aspirin, oral contraceptives, and nonsteroidal anti-inflammatory drugs). The experiments with the in vitro rotation model were performed with heparinized blood of five different volunteers. The other experiments were repeated using the blood of the same volunteer.

### 2.4. Incubation of Blood with Oligonucleotides

To monitor the effect of NU172 on coagulation, citrated or heparinized blood was incubated for 2 min with 0.5, 1.0, 1.5, or 2.0 μM NU172. Then, 1.0 μM AD was added to the blood samples containing NU172. The rheological characteristics of blood and the CT were investigated using the PIEZ system. Furthermore, to validate the results, the CT was additionally measured using a ball coagulometer as a reference device. As controls, blood samples were also analyzed after the addition of 100 µL 0.9% NaCl without oligonucleotides and after the addition of 100 µL 0.9% NaCl containing 1.0 μM NS_AD, NS, or AD. 

### 2.5. Initiation of Coagulation Activation

#### 2.5.1. In Citrated Blood

The coagulation activators pathromtin SL and CaCl_2_ from the activated partial thromboplastin time (aPTT) assay (Siemens Healthcare Diagnostics Products GmbH, Marburg, Germany) were used to activate the coagulation in citrated blood. Therefore, 100 μL pathromtin SL was added to 100 μL citrated blood with and without oligonucleotides and incubated for 2 min at 37 °C. Afterwards, 100 μL CaCl_2_ (c = 0.025 mol/L) was added to initiate the coagulation. The samples (300 μl) were then simultaneously applied onto the measuring surface of PIEZ or into the cuvette of the ball coagulometer to measure the blood coagulation. 

#### 2.5.2. In Heparinized Blood

To initiate the coagulation in heparinized blood, 100 μL heparinized blood with and without oligonucleotides was incubated with the coagulation activators of the Heptest (Heptest Laboratories, Inc, St. Louis, USA). Therefore, 100 μL plasma cephalin was added and incubated for 2 min, and then 100 μL factor X_a_ containing CaCl_2_ was added. A total volume of 300 µL was transferred into the cuvette of the ball coagulometer or onto the measuring surface of the PIEZ. 

### 2.6. Detection of Coagulation

#### 2.6.1. Optimized Piezo-Based Measuring Method (PIEZ)

**Figure 1:**
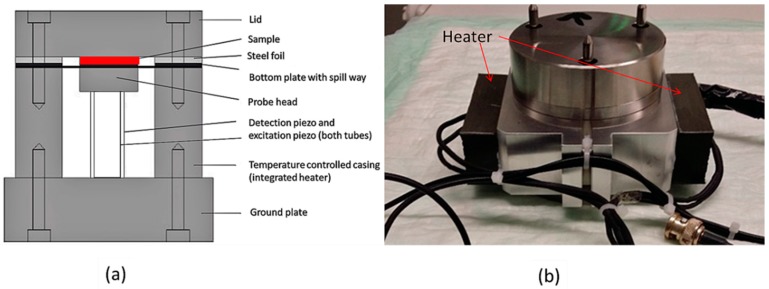
(**a**) Schematic drawing of the optimized PIEZ system (modified after Kirschenmann) [17]. The system consists of a bottom plate and a lid, which hermetically closes the system. The bottom plate is moved by an excitation piezo (inner tube), and the sample is squeezed. The damping of the sample depends on the viscosity of the sample and can be detected by the detection piezo (outer tube). A steel foil creates the measuring gap between the bottom plate and lid. (**b**) Picture of the PIEZ with an integrated heater (indicated by arrows).

To detect the viscoelastic properties of the blood and the CT, the optimized PIEZ system was designed and applied (Figure 1). The lid of the system is fixed to the bottom plate to hermetically close the piezo system. The PIEZ system is connected to a computer and a lock-in amplifier sends the frequency, voltage, and a sinusoidal excitation to the excitation piezo. The bottom plate is moved by the excitation piezo, and a periodical squeeze flow of the sample is created within the measuring chamber. The deformation of the blood sample is measured by the detection of the complex voltage (U*) at the detection piezo and the phase shift between the excitation and detection piezo. Before starting the measurement, a reference reading was performed with an empty device to obtain U_0_*. The system can only differentiate between unloaded and loaded sample measurement if the ratio |U*/U_0_*| is smaller than 1.

The complex shear modulus (G*) is determined by calculating the |U*/U_0_*|ratio. G* consists of a real part, the elastic/storage modulus (G′), and an imaginary part, the viscous/loss modulus (G″). The complex shear viscosity (η*) can be calculated from the shear modulus and consists of a viscous (η′, real part) and an elastic (η″, imaginary part) component. Kirschenmann described the equation of motion of the piezo system in a point-mechanical approximation using a mass-spring system [17]. This approximation allows the correlation between the |U*/U_0_*| and the complex squeeze stiffness (K*). A continuum mechanical calculation is used to obtain a correlation between the K* and η*. Using these calculations, the correlation between the |U*/U_0_*| and η* can be obtained [19,20,21]. 

In the case of linear viscoelasticity, K* can be calculated according to Equation (1).

(1) $$\frac{1}{G^{*}}=\frac{3\pi \;R^{4}}{2\;d^{3}}\frac{\left(1+\frac{\rho \omega^{2}d^{2}}{10\;G^{*}}+\cdots \right)}{K^{*}}$$

In Equation (1), *R* denotes the radius of the plate and *d* is the gap width of the measuring chamber, K* is the complex squeeze stiffness, $\left(1+\frac{\rho \omega^{2}d^{2}}{10\;G^{*}}+\cdots \right)$ is the inertia term, and G* is the complex shear modulus. In this equation, the dependence of G* on geometry (*R* and *d*) is visible [17]. 

The doubling of the radius leads to an approximately 16-fold change in K*. Thus, the increase of the radius allowed the measurement of the blood samples with a measuring gap of 50 µm. The CT, m, and viscous and elastic components of the complex viscosity of the blood samples were determined at 300 s. The measurements were performed with 300 µL blood sample at 37 °C and at a frequency of 100 Hz. Citrated blood samples were activated by adding the coagulation activators pathromtin and CaCl_2_. Heparinized blood was activated by adding plasma cephalin and factor X_a_. The PIEZ system was calibrated prior to measurements using calibration fluids as previously described [19]. 

#### 2.6.2. Ball Coagulometer 

As a reference device, the coagulation was simultaneously determined in 300 µL blood samples using a ball coagulometer (KC 1A, ABW Medizin und Technik GmbH, Germany). Prior to the measurement, the blood sample was filled in a cuvette containing a stainless steel ball and incubated for 2 min at 37 °C. In this coagulometer, a stainless steel ball is kept within a magnetic field. To initiate the coagulation process, citrated blood samples were activated by adding the coagulation activators pathromtin and CaCl_2_. Heparinized blood samples were activated by adding plasma cephalin and factor X_a_. The generation of fibrin threads leads to the displacement of the ball from the magnetic field. The change of position is then detected by a magnetic sensor and thereby, the CT in seconds is determined [22,23]. 

### 2.7. Simulation of ECC Using an in Vitro Rotation Model

To simulate ECC conditions, the aptamer incubation was performed with fresh human whole blood anticoagulated with 1 IU/mL heparin in a dynamic in vitro rotation model. Therefore, polypropylene round-bottom tubes (14 mL, BD Biosciences, New Jersey, USA) were filled with 13 mL blood without oligonucleotides (baseline) or with 1300 µL 0.9% NaCl as control, 1.0 μM NU172, AD, or NS. Additionally, 1.0 μM NU172 was added into heparinized blood and incubated for 2 min. Afterwards, 1.0 μM AD was added to the aptamer containing blood and incubated for 5 min to abrogate the inhibitory effect of the aptamer. The prepared tubes were transferred to a tube rotator (neoLab, Heidelberg, Germany) and incubated at 37 °C for 30 min and 10 rpm. Immediately after the addition of oligonucleotides (0 min) and after 30 min of dynamic incubation, blood samples were collected in tubes containing ethylenediaminetetraacetic acid (1.6 mg mL^−1^, EDTA, Sarstedt, Nümbrecht, Germany) for the detection of cell numbers and FPA. To detect thrombin-antithrombin III (TAT) complex, tubes containing 0.3 mL of citrate solution/3 mL blood and 0.106 M C_6_H_5_Na_3_O_7_ × 2H_2_O (Sartstedt, Nümbrecht, Germany) were used. For β-TG analysis, blood was transferred to 2.7 mL CTAD tubes with 270 μL of 0.109 M CTAD solution containing buffered sodium citrate, theophylline, adenosine, and dipyridamole (BD Vacutainer CTAD, Becton-Dickinson GmbH, Heidelberg, Germany) and stored for 15 min on ice. The EDTA and CTAD preparations were centrifuged at 2500 × g for 20 min at 4 °C, and the citrated blood preparations were centrifuged at 1800 × g for 18 min at RT. The blood plasma of each sample was shock frozen in liquid nitrogen and stored at −80 °C until further investigations. To determine the CT, the coagulation of the blood samples was initiated with plasma cephalin and factor X_a_, and the blood samples were manually loaded into the PIEZ and the ball coagulometer. 

### 2.8. Blood Cell Count Analysis

The number of erythrocytes, leukocytes, and platelets in blood samples was measured using an automated cell count system (ABX Micros 60, HORIBA ABX SAS, Montpellier, France) before and after the incubation in rotation model.

### 2.9. Detection of FPA, TAT, and β-TG

During coagulation, the conversion of prothrombin to thrombin is a key event in the formation of a fibrin clot. The most important coagulation inhibitor is AT neutralizing thrombin by forming a TAT complex. As a result, thrombin irreversibly loses its enzymatic activity. Thus, the TAT concentration in plasma is an indirect marker for the detection of coagulation activation. Another indirect thrombin marker is FPA, which is released whenever thrombin converts fibrinogen into fibrin. Both plasma concentrations serve as indirect markers for the detection of coagulation activation. The platelet marker β-TG is released from α-granules and provides information about the platelet activation [22,23,24].

The level of TAT complexes was determined according to the manufacturer’s instructions using Enzygnost TAT micro enzyme-linked immunosorbent assay (ELISA, Siemens Healthcare Diagnostics Products, Marburg Germany). Furthermore, the amount of β-TG (Asserachrom β-TG, Diagnostica Stago, Asnières sur Seine Cedex, France) and FPA (MyBioSource, Inc. San Diego, USA) were determined in the plasma samples. 

### 2.10. Statistical Analyses

Data are presented as means ± standard deviation (SD). Normally distributed data were analyzed using two-way ANOVA with Bonferroni’s multiple comparison test to determine differences between more than two groups. T-test was performed to compare two groups. Statistical significant differences were defined as *p* < 0.05. The calculations of the mean, SD, and m by regression analysis were performed using Microsoft Excel 2013. Statistical analyses were performed using GraphPad Prism Version 6 (GraphPad Software, La Jolla, San Diego, CA, USA). Diagrams of rheological measurements over time were drawn using Origin Pro 8 (Origin Lab Corporation, Northhampton, USA). 

## 3. Results

### 3.1. Detection of the Coagulation Inhibition in Citrated Blood 

#### 3.1.1. Inhibition of Coagulation in Fresh Human Whole Blood by Addition of NU172 Aptamer 

**Figure 2:**
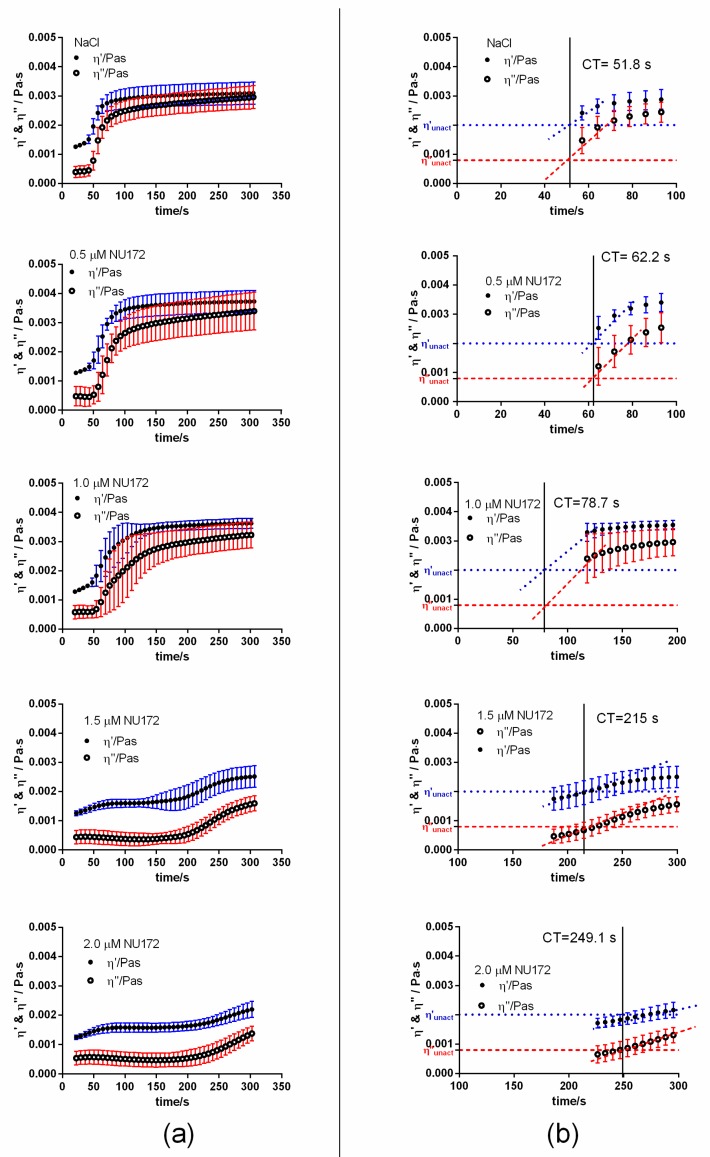
Detection of clotting time (CT) in citrated blood using PIEZ after the addition of 0.5, 1.0, 1.5, or 2.0 µM NU172. Mean viscous (η′) and elastic (η″) components of blood are shown after the addition of (**a**) NaCl (control), 0.5 µM, 1.0 µM, 1.5 µM, or 2.0 µM NU172. The measurements were performed at 100 Hz and 37 °C (n = 10 ± SD). (**b**) Enlarged parts of diagrams for the calculation of CT. The horizontal dotted blue line at 0.002 Pa·s visualizes the η′ and the dashed red line at 0.0008 Pa·s visualizes the η″ of unactivated citrated blood.

To determine the required concentration for the inhibition of coagulation, 0.5, 1.0, 1.5, or 2.0 µM NU172 were added to the citrated blood. Subsequently, the coagulation activation was initiated by the addition of the activators pathromtin and CaCl_2_ from the aPTT assay and the change of η′ and η″ components was detected using PIEZ (Figure 2). 

**Figure 3:**
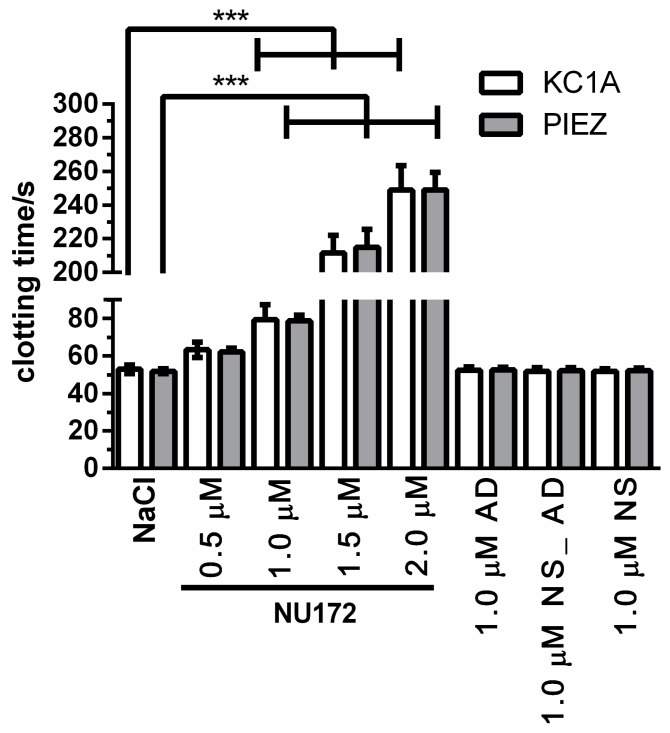
Detection of clotting time (CT) of citrated blood using PIEZ and ball coagulometer (KC 1A) after the addition of 0.5, 1.0, 1.5, or 2.0 µM NU172. Additionally, the CT of blood samples containing NaCI, 1,0 µM AD, NS_AD, or NS were measured. (n = 10 ± SD). The statistical analysis was performed using two-way ANOVA. (*** *p* < 0.001). The CTs measured by KC 1A and PIEZ were not significantly different.

After the activation of coagulation, an increase of viscous (η′) and elastic (η″) components was detected in NaCl containing citrated blood (Figure 2). The linear slopes (m) of the η′ and η″ components at the beginning of the measurement describe the change of the shear viscosity over time and provide information about the dynamics of the coagulation process. Since the CT is an important parameter for the monitoring of blood coagulation and mostly determined by the clinically applied systems, the CT was graphically determined as the intersection between the η′ and η″ of citrated blood without coagulation activation and the m of the η′ and η″ of citrated blood after the activation of coagulation. The addition of increasing concentrations of NU172 (0.5, 1.0, 1.5, or 2.0 µM) to citrated blood prolonged the CT, changed the m of the η′ and η″ and also η′ and η′ values at the end of the measurement (300 s). Increasing of the aptamer concentrations prolonged the CT from 51.8 s (NaCl) to 62.2s (0.5 µM), 78.7 s (1.0 µM), 215 s (1.5 µM), and 249.1 s (2.0 µM). However, the increase of the CT was only significantly higher when 1.0, 1.5, or 2.0 µM NU172 aptamer was added (Figure 3). The incubation of blood with 1.0 µM AD, NS_AD, or NS did not have an effect on the inhibition of thrombin (Figure S1, Table S1). For each oligonucleotide, a CT of approximately 52 s was measured, which is similar to the CT after the addition of NaCl into the blood. Thereby, the aptamer sequence-specific inhibition of coagulation was demonstrated. Furthermore, the CTs observed by PIEZ were comparable to the detected CTs using a ball coagulometer (Figure 3, Table S1). 

#### 3.1.2. Abrogation of NU172 Binding to Thrombin by Addition of AD

**Figure 4:**
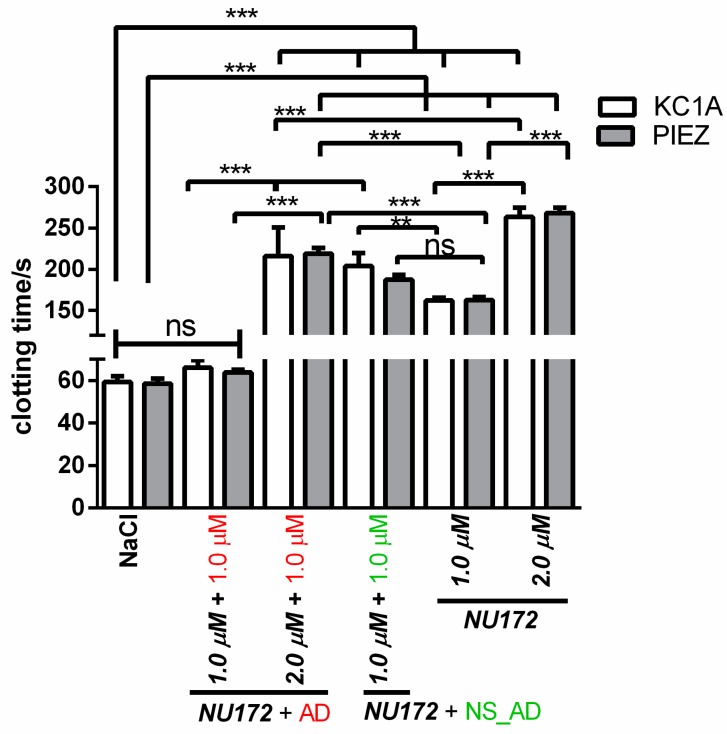
Detection of clotting time (CT) in citrated blood using PIEZ or ball coagulometer (KC1A). After 2 min of incubation with 1.0 or 2.0 µM NU172, 1.0 µM AD or 1.0 µM NS_AD was added. Furthermore, CT was detected in blood containing NaCl, 1.0, or 2.0 µM NU172. Detection of mean viscous (η′) and elastic (η″) components of blood at 100 Hz and 37 °C (n = 5 ± SD). The statistical analysis was performed using two-way ANOVA (*** *p* < 0.001, ** *p* < 0.01, ns: non-significant). The CTs measured by KC 1A and PIEZ were not significantly different.

To analyze the inhibition of NU172 by specific AD, 1.0 µM AD was added to citrated blood without or with 1.0 or 2.0 µM NU172. Furthermore, 1.0 µM NS_AD was added to the blood sample containing 1.0 µM NU172 to show the specific binding of AD. As a positive control, the coagulation of citrated blood with 1.0 or 2.0 µM NU172 was also measured using PIEZ and ball coagulometer (Figure 4, Table S2, Figure S2). The addition of equivalent AD concentration (1.0 µM AD to 1.0 µM NU172 containing blood) led to the reduction of CT from 159 s to 63.8 s, which was comparable to the levels of blood containing NaCl. In contrast, the addition of 1.0 µM AD to 2.0 µM NU172 containing blood was not sufficient to abrogate the binding of all aptamers and to significantly increase the CT. The addition of 1.0 or 2.0 µM NU172 to the blood resulted in an inhibition of coagulation, which was demonstrated by significant prolongation of the coagulation time from 58.6 to 159 s (1.0 µM NU172) and 268.1 s (2.0 µM NU172). The CT of citrated blood with 1.0 µM NU172 + 1.0 µM AD was significantly lower compared to samples containing 1.0 µM NU172 + 1.0 µM NS_AD. Moreover, the blood with 2.0 µM NU172 + 1.0 µM AD exhibited significantly lower CT than blood samples containing 2.0 µM NU172. These results demonstrated that the PIEZ system is capable to measure the coagulation inhibitory effect of NU172 and the abrogation of the aptamer by its complementary AD sequence. Thus, the PIEZ system allows the measurement of CT and the behavior pattern of the viscous (η′) and elastic (η″) components of blood. Currently, no clinical or research system can measure these conditions in such a detailed way. Furthermore, no significant differences in CT were detected between PIEZ and ball coagulometer (Figure 4, Table S2).

Compared to the obtained results in Figure 2 and Figure 3, in this experiment, blood from another volunteer was used, and due to volunteer dependent coagulation activation, both aptamer concentrations led to the detection of a higher CT. Therefore, in this experiment, a CT of 159 s was detected instead of 78.7 s after the addition of 1.0 µM NU172.

### 3.2. Detection of Coagulation Inhibition in Heparinized Blood 

During ECC, such as heart surgery, the patient´s blood is often anticoagulated with heparin. AT activated by heparin binds to the exosite II of thrombin [5], and NU172 binds to exosite I of thrombin. Thus, to analyze whether the influence of the NU172 aptamer on coagulation can be also determined in heparinized blood, 1.0 or 2.0 µM NU172, 1.0 µM NS_AD, or 1.0 µM AD were added to the heparinized blood. After 2 min of incubation, plasma cephalin and factor X_a_ (including CaCl_2_) were added to the blood samples to initiate the coagulation process, and the CT was detected.

**Figure 5:**
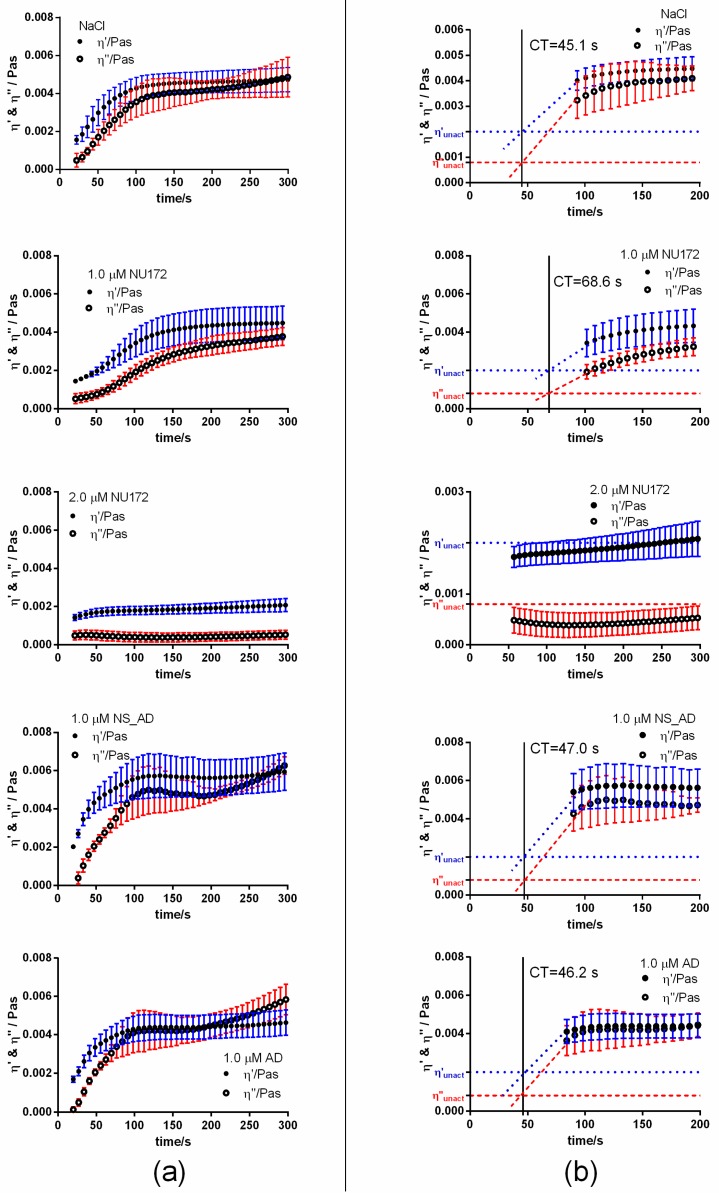
Detection of clotting time (CT) in heparinized blood (1 IU/mL) using PIEZ after the addition of 1.0 or 2.0 µM NU172. Mean viscous (η′) and elastic (η″) components of blood are shown after the addition of (**a**) NaCl (control), 1.0 or 2.0 µM NU172, 1.0 µM NS_AD, or 1.0 µM AD. The measurements were performed at 100 Hz and at 37 °C (n = 10 ± SD). (**b**) Enlarged parts of diagrams for the calculation of CT. The horizontal dotted blue line at 0.002 Pa·s visualizes the η′ and the dashed red line at 0.0008 Pa·s visualizes the η″ of unactivated heparinized blood.

**Figure 6:**
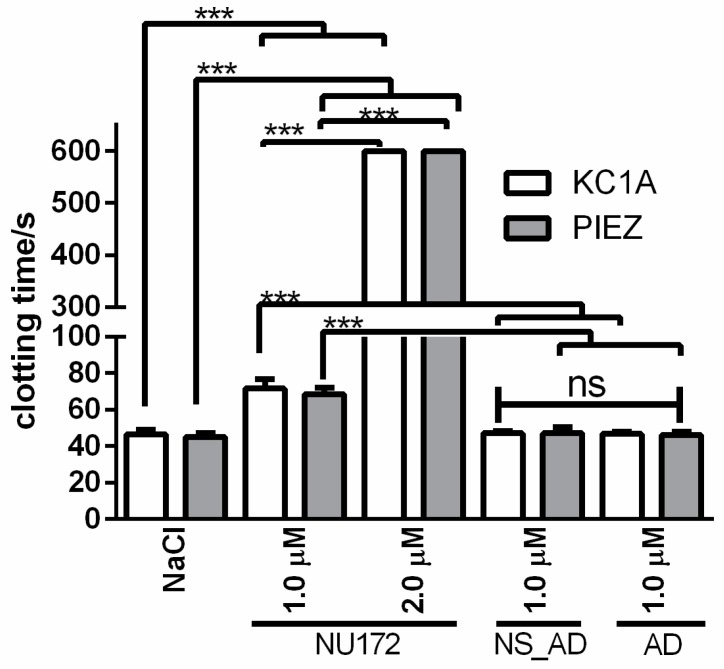
Detection of clotting time (CT) in heparinized blood using PIEZ or ball coagulometer (KC1A). Heparinized blood was incubated for 2 min with 1.0 or 2.0 µM NU172. The coagulation was then activated with cephalin and factor X_a_, and the CT was measured. Furthermore, the CT was detected in blood without oligonucleotide addition (NaCl) or with 1.0 µM NS_AD or AD. The statistical analysis was performed using two-way ANOVA (*** *p* < 0.001). (n = 10 ± SD).

The addition of 1.0 µM NU172 to heparinized blood prolonged the CT from 45.1 s (NaCl) to 68.6 s (Figure 5, Table S3). The addition of negative controls, 1.0 µM NS_AD or AD resulted in CTs, which were comparable to the control heparinized blood containing NaCl. After the addition of 2.0 µM NU172, no clotting was detected during 300 s. The detected CTs were not significantly different between PIEZ and ball coagulometer (Figure 6), which showed that the optimized PIEZ and the reference medical device provide comparable results. Furthermore, the PIEZ visualized the coagulation process by the change of viscous and elastic components over a certain time period. By increasing the NU172 concentration, the CT was prolonged, the slope of the η′ and η″ components, and the values of η′ and η″ components were decreased. Furthermore, it can be concluded that the effect of heparin and NU172 did not affect each other. Both substances together strengthened the inhibitory effect in comparison to the control measurement without aptamer.

### 3.3. Analysis of the Influence of NU172 on Coagulation and the Abrogation of Anticoagulant Effect by Addition of AD Using in Vitro Rotation Model

**Figure 7:**
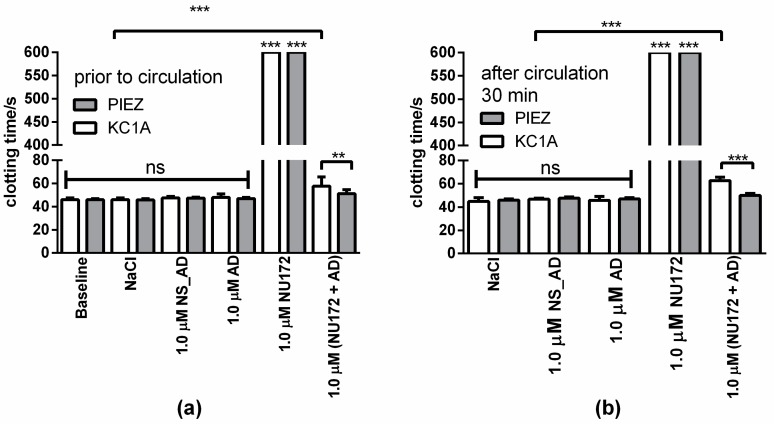
Detection of clotting time (CT) after the dynamic incubation of heparinized blood (1 IU/mL) containing oligonucleotides in an in vitro rotation model using PIEZ system or ball coagulometer (KC1A). Heparinized blood was incubated for 2 min with 1.0 µM NU172, then 1.0 μM AD was added to the NU172 aptamer containing blood and incubated for 5 min. The CT was determined (**a**) before and (**b**) after the circulation for 30 min at 37 °C in an in vitro rotation model. Blood without any addition was indicated as a baseline. Blood with NaCl, 1.0 µM NS_AD, or AD was used as a negative control. Blood with 1.0 µM NU172 served as a positive control. Blood from five different volunteers was used (n = 5 ± SD). The statistical analysis was performed using two-way ANOVA (** *p* < 0.01, *** *p* < 0.001, ns: not significant).

Flow conditions similar to ECC were simulated using an in vitro rotation model and heparinized blood (1 IU/mL). Therefore, 1.0 µM NU172, AD, or NS_AD were added into heparinized blood and incubated for 2 min. Furthermore, blood without oligonucleotides (baseline) or with NaCl was used as control. Then, 1.0 μM AD was added to the NU172 aptamer containing blood and incubated for 5 min to abrogate the inhibitory effect. A blood sample with 1.0 µM NU172 but without AD was also used as a positive control. The CT was determined before and after the circulation for 30 min at 37 °C using the PIEZ system and ball coagulometer (Figure 7, Table S4). The addition of 1.0 µM NU172 significantly prolonged the CT form approximately 46 s (NaCl containing blood) to over 600 s. In this experiment, a volunteer dependent increased CT was detected compared to the obtained CT values during the static analysis of aptamer mediated coagulation inhibition in heparinized blood (Figure 5 and Figure 6). The addition of 1.0 µM AD, NS_AD, or 1.0 µM NU172 + 1.0 µM AD resulted in comparable CTs as the blood samples containing NaCl. These results demonstrated that the negative control oligonucleotides (AD or NS_AD) did not have an influence on coagulation. 

The detected CTs using PIEZ and KC 1A were not significantly different from each other, except for 1.0 µM (NU172 + AD) before and after the circulation (Figure 7). The ball coagulometer provided for blood samples containing 1.0 µM (NU172 + AD) slightly higher CTs than the PIEZ system. Furthermore, using a ball coagulometer, slightly higher CTs were also detected in blood samples with 1.0 µM (NU172+ AD) (both time points) than in the control (NaCl), which indicates a remaining amount of NU172 that was not complexed with AD. In accordance with our previous publication [7], already after 5 min of incubation with the AD, the anticoagulation effect of NU172 was abolished and maintained during the dynamic incubation in the in vitro rotation model of 30 min. 

**Figure 8:**
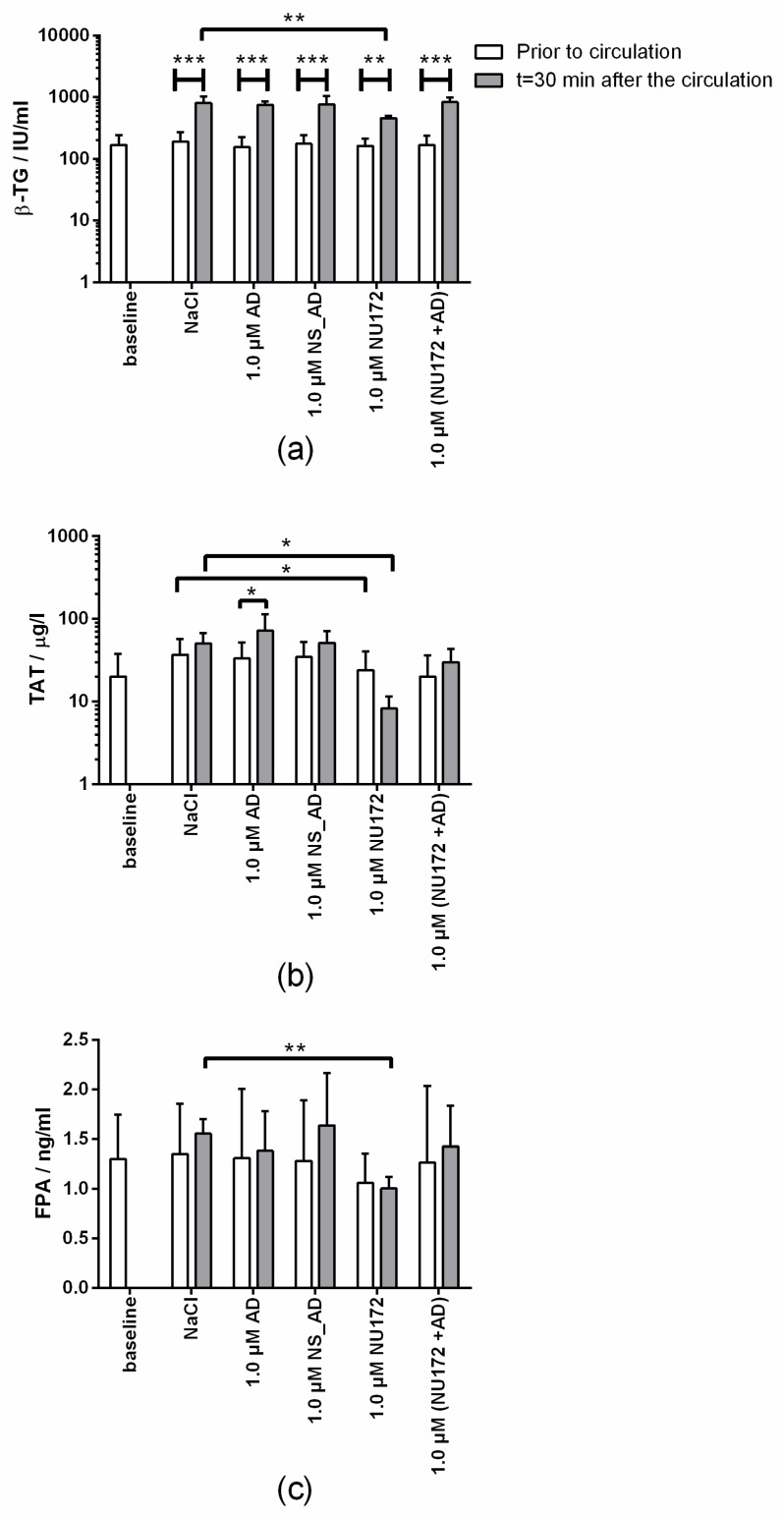
Determination of coagulation activation markers. Heparinized blood with 1.0 µM NU172 or 1.0 µM NU172 and 1.0 µM AD was incubated for 0 and 30 min at 37 °C in an in vitro rotation model. Blood without any addition was indicated as a baseline. Using ELISA, the concentrations of (**a**) β-TG, (**b**) TAT and (**c**) FPA were determined before and after the circulation. Blood from five different volunteers was used (n = 5 ± SD). The statistical analysis was performed using t-test (* *p* < 0.05, ** *p* < 0.01, *** *p* < 0.001).

Additionally to the CT, the thrombin markers (FPA, TAT, and β-TG) (Figure 8) were analyzed. In all blood samples, β-TG values were increased after the circulation, which showed that only the circulation of the blood resulted in increased activation of platelets (Figure 8a). After 30 min of circulation, the blood samples containing 1.0 µM NU172 showed a significantly reduced TAT, FPA, and β-TG concentrations compared to the NaCl containing blood. These results were consistent with the detected inhibition of blood coagulation measured by PIEZ. Compared to the blood samples without circulation, the blood samples containing 1.0 µM AD showed a slightly increased TAT concentration after 30 min of circulation.

**Figure 9:**
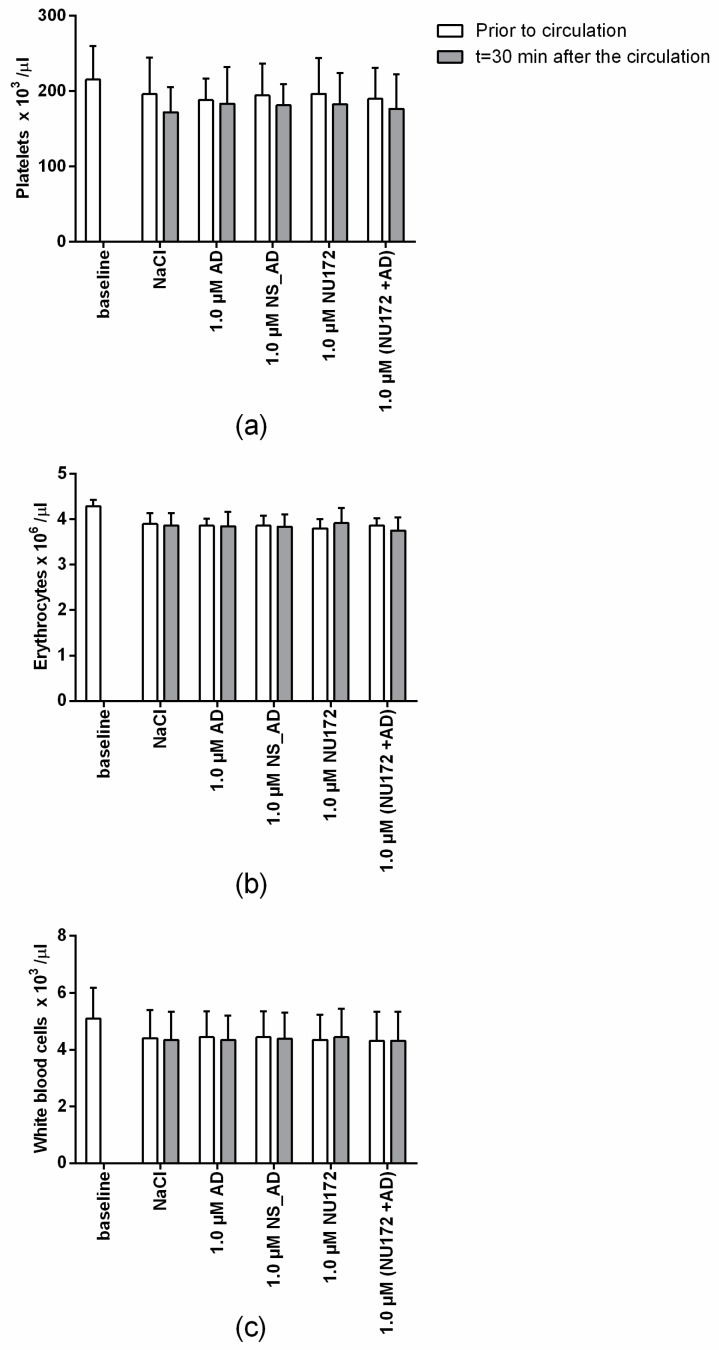
Blood cell count analysis of heparinized blood before and after the incubation in an in vitro rotation model. Heparinized blood was incubated for 30 min at 37 °C with 1.0 µM NU172 or 1.0 µM NU172 and 1.0 µM AD. Blood without any addition was indicated as a baseline. (**a**) Platelets, (**b**) erythrocytes, and (**c**) white blood cell counts are shown (n = 5 ± SD).

The circulation in the in vitro rotation model can lead to an activation of thrombocytes, hemolysis, or attachment of the white blood cells to artificial surfaces. This can then result in a decrease in the numbers of thrombocytes, erythrocytes, or/and leukocytes. Thus, we additionally analyzed the blood cell numbers before and after the circulation of blood in the rotation model (Figure 9) and no significant differences in platelets, erythrocytes, and white blood cell counts were detected compared to the baseline. Furthermore, the results also showed that the addition of thrombin aptamer had no influence on blood cell numbers.

## 4. Discussion

In this study, a new optimized PIEZ was developed to evaluate the blood coagulation status by detecting the rheological characteristics of the sample. The sensitivity for blood rheology measurements was optimized by doubling the diameter of the measuring chamber and reducing the mechanical stiffness of the former PAV system. Using the new PIEZ, the influence of thrombin binding aptamer NU172 on coagulation inhibition could be determined in citrated as well as heparinized blood samples. The CTs obtained by PIEZ measurements were comparable to those measured with the medical reference system, the ball coagulometer KC 1A. As an additional feature, PIEZ allowed the detection of slope m of viscous (η′) and elastic (η″) components, which describe the kinetics of the coagulation process as a change of shear viscosity over time. The detection of shear viscosity (η′ and η″ components) at a given time provides information about the proportion of each component in the sample. In addition, the detection of shear viscosity 300 s after the initiation of coagulation gives an indication of the rigidity of the resulting blood clot. 

At present, simple (e.g., ball coagulometer (KC 1A)) or sophisticated devices (e.g. rotational thromboelastometry (ROTEM)) are clinically used to analyze blood coagulation. Currently, new devices are being developed, such as cavitation rheology, which investigates the rheological properties of erythrocytes [25,26]. Thereby, a pulsed laser with a wavelength of 532 nm is focused on the sample for 6 ns. Due to the high intensities at the focal volume, an expanding bubble, called cavity, is formed. The bubbles reach a diameter of 90–120 µm before collapsing. The elastic properties of erythrocytes can be characterized by analyzing the shape recovery of the cells. Thus, this method can be applied to study the properties of blood cells, but it is not suitable for the monitoring of hemostasis. In contrast, the viscoelastic hemostatic assay, free oscillation rheology (FOR) might be a better suited rheological method for the detection of blood coagulation [27,28]. To obtain the CT, citrated blood or plasma is added at 37 °C to a free oscillating cuvette. A magnetic field initiates the oscillation every 2 s with a frequency of 11 Hz. An optical detector registers the damping and frequency of the container. During the coagulation process, the damping increases and the frequency decreases. Therefore, the endpoint of coagulation can be detected by a change in elasticity. This system is similar to thromboelastography (TEG) and ROTEM, but FOR is not a widely used method in investigations of coagulopathy during ECC [29]. 

ROTEM is a medical device that is used in clinical practice [30,31,32]. It uses a fixed cuvette (cup) with a cylindrical pin that is immersed into the cup filled with whole blood (gap of 1 mm between pin and cuvette wall). The movement of the rotating pin is restricted once the blood starts clotting. A spring affixed to the pin detects the restriction. The kinetic change is optically detected by an integrated computer (TEMogram). Using multiple cuvettes simultaneously with different reagents, the interactions of coagulation factors, inhibitors, and cellular components can be measured during the coagulation phase as well as subsequent fibrinolysis over time [31,33,34,35]. Currently, ROTEM remains the gold standard for sophisticated hemostasis monitoring [29]. The first generation device, ROTEM delta is semi-automated with an automatic pipette system and manual connection of the pin-and-cup system. It requires limited manual training, special expertise in coagulation and extensive interpretation training. Especially, the first generation TEG, TEG 5000, is associated with extensive manual handling and shock-sensitivity. Thus, this device cannot be used at the patient´s bedside as it requires a low-vibration workstation. To solve these challenges, ROTEM sigma and TEG 6S were designed. These devices are cartridge-based, fully automated with preset reagents, no manual pipetting steps are required, and have decreased sensitivity to external vibrations. However, special extensive expertise is required for the interpretation of the results and the devices are expensive. 

The new PIEZ in this study can monitor within five minutes the change of viscous and elastic components over time and provides reliable information about the blood coagulation. Furthermore, the piezo-based system is able to measure the coagulation status in real-time. Additionally, the PIEZ system is not susceptible to shock, which prevents disturbance of measurements and increases the reproducibility of results. Currently, the PIEZ system is a research instrument, and since it needs manual cleaning after each measurement, it is not applicable during ECC. However, after further improvement and optimization, the system might be also used at the patient´s bedside for continuous monitoring. The shear viscosity measured at a given time and the change in shear viscosity over time (m) using PIEZ system could be comparable to other viscoelastic monitoring devices, such as FOR [36] or ROTEM [30]. In further studies, PIEZ will be also compared with these devices. For further applications at the operation theater, hardware improvements and miniaturization of the PIEZ system are necessary. For example, a disposable measuring chamber could be created as a cuvette or using a microfluidic system, the measuring chamber could be connected to the ECC system for the continuous monitoring of the coagulation. In addition, a cleaning and waste reservoir could be integrated into the microfluidic system, and the pipetting steps could be minimized by automatization. 

Aptamers are promising drug candidates with the ability to bind to specific targets, such as cells or proteins (growth factors, transcription factors, enzymes, immunoglobulins, and receptors) with high specificity and affinity and have the ability to modulate the targets’ activities [11,37,38]. In this study, using the new PIEZ system, we were able to reliably measure the coagulation inhibiting effect of the thrombin binding aptamer NU172 after static and dynamic incubation and the ability of its complementary antidote (AD) to abrogate the coagulation inhibitory effect of NU172. In our previous study, the required time for the generation of double-stranded complexes was examined [7], and it was found that the AD was able to bind already after 2 min to the NU172 aptamer, which resulted in rapid abrogation of the anticoagulant activity of NU172 after 5 min in whole blood. 

The addition of 1.0 µM NU172 into the blood led to prolonged CT and decreased m, η′, and η″ compared to the blood samples without oligonucleotide addition. Using PIEZ and different NU172 concentrations, the required aptamer concentration for efficient inhibition of coagulation could be determined. The in vitro rotation experiments with heparinized blood showed that the coagulation can be further inhibited by the addition of NU172 aptamer. Since the anticoagulant effect of aptamers can be rapidly interrupted by a corresponding AD, these aptamers are potent candidates for the applications during extracorporeal circulation in combination with a reduced heparin concentration or for substitution of heparin by a sufficient amount of aptamer in heparin-induced thrombocytopenia (HIT) patients. 

In summary, the established PIEZ system is able to provide information in the form of CT, clot formation (m(η′), m(η″)), and clot firmness (η′and η″ at 300 s). The in vitro rotation experiments and the subsequent ELISA measurements confirmed the results obtained by using PIEZ and KC 1A. The addition of 1.0 µM NU172 into heparinized blood and the incubation at 37 °C for 30 min resulted in a significant reduction of β-TG, TAT, and FPA amounts compared to blood samples without NU172 addition. In this study, using the in vitro rotation model and the comparison with the ball coagulometer, a proof of concept was provided that the PIEZ can be used to analyze blood coagulation. In the next studies, we will perform blood coagulation analyses during a simulated ECC with a heart-lung machine and plan to optimize the system for automated blood sampling and blood coagulation analysis at defined time intervals. Furthermore, we aim to compare the PIEZ device with more sophisticated medical reference systems such as ROTEM or TEG.

## 5. Conclusions

In summary, the results of this study provided a proof-of-concept that the optimized PIEZ system can be used to analyze blood clotting in combination with nucleic acid-based drugs. The device allows a reliable monitoring of coagulation in citrated, as well as heparinized, blood. In addition, using the PIEZ, the functionality of ADs for the inhibition of anticoagulant aptamers can be analyzed. However, to allow the POC application of the PIEZ system, further studies and optimizations are necessary regarding automatization and continuous monitoring of the hemostasis.

## Figures and Tables

**Table 1:** List of used oligonucleotides.

Name	Length (Nucleotides)	Sequence of Oligonucleotides 5′ → 3′
NU172	26	CGCCTAGGTTGGGTAGGGTGGTGGCG
NS ^1^	26	CATCAGTTACATGCACTATCAGTACT
AD ^2^	26	CGCCACCACCCTACCCAACCTAGGCG
NS_AD	26	AGTACTGATAGTGCATGTAACTGATG

^1^ NS: nonsense; ^2^ AD: antidote.

## Supplementary Materials

The following are available online at https://www.mdpi.com/1424-8220/20/1/152/s1, Figure S1: Detection of clotting time (CT) in citrated blood using PIEZ after the addition of 1.0 µM AD, NS, or NS_AD. (a) Detection of mean viscous (η′) and elastic (η″) components of blood at 100 Hz and 37 °C (n = 10 ± SD). (b) Enlarged parts of diagrams for the calculation of CT. The horizontal dotted blue line at 0.002 Pa·s visualizes the η′ and the dashed red line at 0.0008 Pa·s visualizes the η″ of unactivated citrated blood. Figure S2: Detection of clotting time (CT) in citrated blood using PIEZ. After 2 min of incubation with 1.0 or 2.0 µM NU172, 1.0 µM AD or 1.0 µM NS_AD was added. Furthermore, CT was detected in blood containing NaCl, 1.0, or 2.0 µM NU172. (**a**) Detection of mean viscous (η′) and elastic (η″) components of blood at 100 Hz and 37 °C (n = 5 ± SD). (**b**) Enlarged parts of diagrams for the calculation of CT. The horizontal dotted blue line at 0.002 Pa·s visualizes the η′ and the dashed red line at 0.0008 Pa·s visualizes the η″ of unactivated citrated blood. Table S1: Citrated blood was incubated with NaCl or various concentrations of NU172 aptamer, AD, or NS_AD for 2 min. Afterwards, the coagulation was activated by pathromtin and CaCl_2_ (n = 10) and measured with PIEZ and KC 1A. Using PIEZ, the clotting time (CT), viscous (η′) and elastic (η″) components at 300 s, and linear slopes (m(η′) and m(η″)) were measured. Table S2: Citrated blood was incubated with NaCl or various concentrations of NU172 aptamer for 2 min. Then, 1.0 µM AD was added and after 5 min the blood samples were activated with pathromtin and CaCl_2_. (n = 5). The measurement was performed with PIEZ and KC 1A. Using PIEZ, the clotting time (CT), viscous (η′) and elastic (η″) components at 300 s, and linear slopes (m(η′) and m(η″)) were measured. Table S3: Heparinized blood was incubated with NaCl, NU172 aptamer, NS_AD, or AD. After 2 min of incubation, the coagulation was activated with plasma cephalin and factor X_a_. (n = 10). The measurement was performed with PIEZ and KC 1A. Using PIEZ, the clotting time (CT), viscous (η′) and elastic (η″) components at 300 s, and linear slopes (m(η′) and m(η″)) were measured. Table S4: Heparinized blood was incubated for 2 min with 1.0 µM NU172, then 1.0 μM AD was added to the NU172 aptamer containing blood and incubated for 5 min. The clotting time (CT) was determined before and after the circulation for 30 min at 37 °C in an in vitro rotation model using PIEZ or KC 1A. Blood without any additives was indicated as a baseline. Blood with NaCl, 1.0 µM NS_AD, or AD were used as negative controls. Blood with 1.0 µM NU172 served as positive control. Blood from five different volunteers was used (n = 5 ± SD). Using PIEZ, the clotting time (CT), viscous (η′) and elastic (η″) components at 300 s, and linear slopes (m(η′) and m(η″)) were measured.
