# Supplementary material for: Application of Piezo-Based Measuring System for Evaluation of Nucleic Acid-Based Drugs Influencing the Coagulation
Source: Sensors (Basel). 2019 Dec 25;20(1):152. doi: 10.3390/s20010152 (PMC6982813; doi:10.3390/s20010152)
Supplement: Supplementary file 1 [file sensors-20-00152-s001.pdf]

Supplementary

Application of Piezo-Based Measuring System for Evaluation of Nucleic Acid-Based Drugs Influencing the Coagulation

Silju-John Kunnakattu [1,2], Ludmilla Hann [1], Julia Kurz [1], Hanna Haag [1], Stefan Fennrich [1], Nicole Rauch [2], Christian Schlensak [1], Hans Peter Wendel [1], Sandra Stoppelkamp [1,†], and Meltem Avci-Adali [1,*,†]

[1] Department of Thoracic and Cardiovascular Surgery, University Hospital Tuebingen, 72076 Tuebingen, Germany; Silju.kunnakattu@klinikum.uni-tuebingen.de (S.-J.K.); Ludmilla.hann@klinikum.uni-tuebingen.de (L.H.); Julia.kurz@klinikum.uni-tuebingen.de (J.K.); Hanna.haag@klinikum.uni-tuebingen.de (H.H.); stefan.fennrich@gmail.com (S.F.); Christian.schlensak@med.uni-tuebingen.de (C.S.); Hans-peter.wendel@med.uni-tuebingen.de (H.P.W.); Sandra.stoppelkamp@klinikum.uni-tuebingen.de (S.S), meltem.avci-adali@uni-tuebingen.de (M.A.-A.)

[2] Department of Micro- and Nanoanalytics, University of Applied Sciences Iserlohn, 58644 Iserlohn, Germany; Rauch.nicole@fh-swf.de (N.R.)

* Correspondence: meltem.avci-adali@uni-tuebingen.de ; Tel.: +49-7071-29-83334; Fax: + 49-7071-29-5369

† These authors contributed equally to this work.

Supplementary Tables

Table S1. Citrated blood was incubated with NaCl or various concentrations of NU172 aptamer, AD, or NS_AD for 2 min. Afterwards, the coagulation was activated by pathromtin and $CaCl_2$ (n = 10) and measured with PIEZ and KC 1A. Using PIEZ, the clotting time (CT), viscous (η′) and elastic (η″) components at 300 s, and linear slopes (m(η′) and m(η″)) were measured [1].

Sample	PIEZ	KC 1A	PIEZ			
	CT/s	CT/s	η′(Pas) at 300 s	η″(Pas) at 300 s	m(η′/Pa)	m(η″/Pa)
**NaCl**	51.8 ± 1.3	52.9 ± 2.3	$3.1 \times 10^{-3} \pm 3.8 \times 10^{-4}$	$3.0 \times 10^{-3} \pm 3.9 \times 10^{-4}$	$6.8 \times 10^{-5} \pm 1.2 \times 10^{-5}$	$8.4 \times 10^{-5} \pm 1.2 \times 10^{-5}$
**0.5 µM NU172**	62.2 ± 1.9	63.3 ± 4.0	$3.7 \times 10^{-3} \pm 3.7 \times 10^{-4}$	$3.4 \times 10^{-3} \pm 6.1 \times 10^{-4}$	$7.8 \times 10^{-5} \pm 1.6 \times 10^{-5}$	$6.8 \times 10^{-5} \pm 2.2 \times 10^{-5}$
**1.0 µM NU172**	78.7 ± 3.1	79.4 ± 8.0	$3.5 \times 10^{-3} \pm 2.6 \times 10^{-4}$	$3.2 \times 10^{-3} \pm 3.8 \times 10^{-4}$	$4.9 \times 10^{-5} \pm 1.3 \times 10^{-5}$	$4.2 \times 10^{-5} \pm 2.2 \times 10^{-5}$
**1.5 µM NU172**	215.0 ± 10.7	211.6 ± 10.4	$2.5 \times 10^{-3} \pm 3.6 \times 10^{-4}$	$1.6 \times 10^{-3} \pm 2.6 \times 10^{-4}$	$1.1 \times 10^{-5} \pm 3.5 \times 10^{-6}$	$1.4 \times 10^{-5} \pm 2.5 \times 10^{-6}$
**2.0 µM NU172**	249.1 ± 10.5	248.9 ± 14.6	$2.1 \times 10^{-3} \pm 2.8 \times 10^{-4}$	$1.4 \times 10^{-3} \pm 2.4 \times 10^{-4}$	$8.3 \times 10^{-6} \pm 3.1 \times 10^{-6}$	$1.2 \times 10^{-5} \pm 2.5 \times 10^{-6}$
**1.0 µM AD**	52.7 ± 1.1	52.4 ± 1.9	$2.9 \times 10^{-3} \pm 2.0 \times 10^{-4}$	$3.5 \times 10^{-3} \pm 4.6 \times 10^{-4}$	$5.9 \times 10^{-5} \pm 6.7 \times 10^{-6}$	$9.1 \times 10^{-5} \pm 1.2 \times 10^{-5}$
**1.0 µM NS_AD**	52.2 ± 1.6	51.8 ± 1.9	$3.3 \times 10^{-3} \pm 2.8 \times 10^{-4}$	$3.5 \times 10^{-3} \pm 4.1 \times 10^{-4}$	$8.0 \times 10^{-5} \pm 1.0 \times 10^{-5}$	$9.9 \times 10^{-5} \pm 2.7 \times 10^{-5}$
**1.0 µM NS**	52.1 ± 1.6	51.8 ± 1.5	$3.3 \times 10^{-3} \pm 2.9 \times 10^{-4}$	$3.4 \times 10^{-3} \pm 4.0 \times 10^{-4}$	$8.2 \times 10^{-5} \pm 1.2 \times 10^{-5}$	$1.1 \times 10^{-4} \pm 2.4 \times 10^{-5}$

[1.] clotting time (CT), viscous component (η′), elastic component (η″), the linear slope of the viscous or elastic component (m), thrombin aptamer (NU172), antidote (AD), nonsense antidote (NS_AD), nonsense (NS).

Table S2. Citrated blood was incubated with NaCl or various concentrations of NU172 aptamer for 2 min. Then, 1.0 μM AD was added and after 5 min the blood samples were activated with pathromtin and $CaCl_2$. (n = 5). The measurement was performed with PIEZ and KC 1A. Using PIEZ, the clotting time (CT), viscous (η′) and elastic (η″) components at 300 s, and linear slopes (m(η′) and m(η″)) were measured [1].

Sample	PIEZ	KC 1A	PIEZ			
	CT/s	CT/s	η′(Pas) at 300 s	η″(Pas) at 300 s	m(η′/Pa)	m(η″/Pa)
**NaCl**	58.6 ± 2.4	59.4 ± 2.5	$3.3 \times 10^{-3} \pm 3.0 \times 10^{-4}$	$3.6 \times 10^{-3} \pm 1.4 \times 10^{-4}$	$7.2 \times 10^{-5} \pm 1.1 \times 10^{-5}$	$8.6 \times 10^{-5} \pm 3.6 \times 10^{-5}$
**NU172**						
**1.0 μM**	162.6 ± 3.6	162.0 ± 3.3	$3.3 \times 10^{-3} \pm 3.1 \times 10^{-4}$	$2.0 \times 10^{-3} \pm 3.5 \times 10^{-4}$	$2.1 \times 10^{-5} \pm 3.5 \times 10^{-6}$	$1.9 \times 10^{-5} \pm 2.1 \times 10^{-6}$
**2.0 μM**	268.1 ± 6.6	263.0 ± 11.5	$2.4 \times 10^{-3} \pm 1.9 \times 10^{-4}$	$1.3 \times 10^{-3} \pm 2.2 \times 10^{-4}$	$1.2 \times 10^{-5} \pm 8.5 \times 10^{-7}$	$1.3 \times 10^{-5} \pm 2.6 \times 10^{-6}$
**NU172+AD**						
**1.0 μM+1.0 μM**	63.8 ± 1.4	66.1 ± 3.4	$3.0 \times 10^{-3} \pm 1.7 \times 10^{-4}$	$3.3 \times 10^{-3} \pm 7.5 \times 10^{-4}$	$5.6 \times 10^{-5} \pm 5.7 \times 10^{-6}$	$7.1 \times 10^{-5} \pm 1.1 \times 10^{-5}$
**2.0 μM+1.0 μM**	218.6 ± 7.3	215.7 ± 34.7	$2.7 \times 10^{-3} \pm 2.4 \times 10^{-4}$	$2.2 \times 10^{-3} \pm 4.6 \times 10^{-4}$	$9.3 \times 10^{-6} \pm 3.4 \times 10^{-6}$	$1.8 \times 10^{-5} \pm 2.0 \times 10^{-5}$
**NU172+NS_AD**						
**1.0 μM+1.0 μM**	187.3 ± 5.8	203.7 ± 15.8	$2.6 \times 10^{-3} \pm 1.9 \times 10^{-4}$	$2.7 \times 10^{-3} \pm 4.9 \times 10^{-4}$	$8.9 \times 10^{-6} \pm 6.2 \times 10^{-6}$	$1.9 \times 10^{-5} \pm 7.8 \times 10^{-6}$

[1.] clotting time (CT), viscous component (η′), elastic component (η″), the linear slope of the viscous or elastic component (m), thrombin aptamer (NU172), antidote (AD), nonsense antidote (NS_AD).

Table S3. Heparinized blood was incubated with NaCl, NU172 aptamer, NS_AD, or AD. After 2 min of incubation, the coagulation was activated with plasma cephalin and factor X_a. (n = 10). The measurement was performed with PIEZ and KC 1A. Using PIEZ, the clotting time (CT), viscous (η′) and elastic (η″) components at 300 s, and linear slopes (m(η′) and m(η″)) were measured [1].

Sample	PIEZ	KC 1A	PIEZ			
	CT/s	CT/s	η′(Pa s) at 300 s	η″ (Pa s) at 300 s	m(η′/Pa)	m(η″/Pa)
**NaCl**	45.1 ± 2.1	46.6 ± 2.3	$4.6 \times 10^{-3} \pm 5.2 \times 10^{-4}$	$4.6 \times 10^{-3} \pm 9.2 \times 10^{-4}$	$4.0 \times 10^{-5} \pm 8.2 \times 10^{-6}$	$3.8 \times 10^{-5} \pm 1.6 \times 10^{-5}$
**1.0 μM NU172**	68.6 ± 3.4	71.7± 4.9	$4.4 \times 10^{-3} \pm 8.4 \times 10^{-4}$	$3.6 \times 10^{-3} \pm 8.0 \times 10^{-4}$	$2.1 \times 10^{-5} \pm 1.4 \times 10^{-6}$	$2.1 \times 10^{-5} \pm 5.5 \times 10^{-6}$
**2.0 μM NU172**	>600	>600	$1.9 \times 10^{-3} \pm 2.4 \times 10^{-4}$	$5.4 \times 10^{-3} \pm 2.3 \times 10^{-4}$	$3.4 \times 10^{-6} \pm 3.1 \times 10^{-6}$	$1.1 \times 10^{-6} \pm 3.1 \times 10^{-5}$
**1.0 μM NS_AD**	47.0± 3.0	47.1± 1.3	$5.6 \times 10^{-3} \pm 1.0 \times 10^{-3}$	$6.0 \times 10^{-3} \pm 7.8 \times 10^{-4}$	$2.8 \times 10^{-5} \pm 1.3 \times 10^{-5}$	$4.9 \times 10^{-5} \pm 1.6 \times 10^{-5}$
**1.0 μM AD**	46.2± 1.8	46.8± 1.3	$4.5 \times 10^{-3} \pm 6.1 \times 10^{-4}$	$5.8 \times 10^{-3} \pm 7.6 \times 10^{-4}$	$3.0 \times 10^{-5} \pm 3.3 \times 10^{-6}$	$4.3 \times 10^{-5} \pm 1.4 \times 10^{-5}$

[1.] clotting time (CT), viscous component (η′), elastic component (η″), the linear slope of the viscous or elastic component (m), thrombin aptamer (NU172), antidote (AD), nonsense antidote (NS_AD)

Table S4. Heparinized blood was incubated for 2 min with 1.0 µM NU172, then 1.0 µM AD was added to the NU172 aptamer containing blood and incubated for 5 min. The clotting time (CT) was determined before and after the circulation for 30 min at 37 °C in an in vitro rotation model using PIEZ or KC 1A. Blood without any additives was indicated as a baseline. Blood with NaCl, 1.0 µM NS_AD, or AD were used as negative controls. Blood with 1.0 µM NU172 served as positive control. Blood from five different volunteers was used (n = 5 ± SD). Using PIEZ, the clotting time (CT), viscous (η′) and elastic (η″) components at 300 s, and linear slopes (m(η′) and m(η″)) were measured [1].

**Sample**	**PIEZ**	**KC 1A**	**PIEZ**			
	**CT/s**	**CT/s**	**η′(Pa s) at 300 s**	**η″ (Pa s) at 300 s**	**m(η′/Pa)**	**m(η″/Pa)**
**Baseline**	45.9 ± 1.2	45.7 ± 2.0	$8.1 \times 10^{-3} \pm 3.8 \times 10^{-3}$	$1.2 \times 10^{-2} \pm 4.0 \times 10^{-3}$	$5.7 \times 10^{-5} \pm 2.0 \times 10^{-5}$	$3.4 \times 10^{-4} \pm 3.3 \times 10^{-4}$
**0 min**						
**NaCl**	45.5± 1.2	45.7± 2.0	$5.7 \times 10^{-3} \pm 2.6 \times 10^{-4}$	$9.4 \times 10^{-3} \pm 2.5 \times 10^{-3}$	$4.8 \times 10^{-5} \pm 5.4 \times 10^{-6}$	$1.8 \times 10^{-4} \pm 8.2 \times 10^{-5}$
**1.0 µM NS_AD**	47.6 ± 0.9	47.2 ± 1.8	$8.2 \times 10^{-3} \pm 1.1 \times 10^{-3}$	$1.0 \times 10^{-2} \pm 3.1 \times 10^{-3}$	$8.9 \times 10^{-5} \pm 2.8 \times 10^{-5}$	$2.7 \times 10^{-4} \pm 8.1 \times 10^{-5}$
**1.0 µM AD**	46.7± 1.3	47.7± 4.2	$7.0 \times 10^{-3} \pm 3.6 \times 10^{-3}$	$1.0 \times 10^{-2} \pm 5.5 \times 10^{-3}$	$7.5 \times 10^{-5} \pm 5.2 \times 10^{-5}$	$2.3 \times 10^{-4} \pm 1.6 \times 10^{-4}$
**1.0 µM NU172**	> 600	> 600	$4.2 \times 10^{-3} \pm 5.6 \times 10^{-4}$	$2.2 \times 10^{-3} \pm 9.0 \times 10^{-4}$	$1.1 \times 10^{-5} \pm 3.2 \times 10^{-6}$	$8.1 \times 10^{-6} \pm 1.9 \times 10^{-6}$
**1.0 µM NU172 + 1.0 µM AD**	51.9± 4.6	57.9. ± 11.4	$5.7 \times 10^{-3} \pm 4.6 \times 10^{-4}$	$7.3 \times 10^{-3} \pm 7.7 \times 10^{-4}$	$3.9 \times 10^{-5} \pm 2.0 \times 10^{-5}$	$1.2 \times 10^{-4} \pm 7.2 \times 10^{-5}$
**After 30 min**						
**NaCl**	45.3 ± 0.9	44.0 ± 4.2	$4.5 \times 10^{-3} \pm 1.6 \times 10^{-3}$	$6.7 \times 10^{-3} \pm 3.3 \times 10^{-3}$	$3.1 \times 10^{-5} \pm 1.5 \times 10^{-5}$	$8.7 \times 10^{-5} \pm 5.2 \times 10^{-5}$
**1.0 µM NS_AD**	47.7 ± 1.4	46.4 ± 1.2	$6.1 \times 10^{-3} \pm 1.5 \times 10^{-3}$	$8.3 \times 10^{-3} \pm 1.2 \times 10^{-3}$	$5.2 \times 10^{-5} \pm 3.4 \times 10^{-5}$	$1.4 \times 10^{-4} \pm 1.1 \times 10^{-4}$
**1.0 µM AD**	46.7 ± 1.6	45.3 ± 4.9	$7.8 \times 10^{-3} \pm 3.4 \times 10^{-3}$	$9.9 \times 10^{-3} \pm 4.4 \times 10^{-3}$	$8.8 \times 10^{-5} \pm 6.0 \times 10^{-5}$	$2.5 \times 10^{-4} \pm 1.6 \times 10^{-4}$
**1.0 µM NU172**	> 600	> 600	$4.4 \times 10^{-3} \pm 5.5 \times 10^{-4}$	$2.3 \times 10^{-2} \pm 9.9 \times 10^{-4}$	$1.5 \times 10^{-5} \pm 2.6 \times 10^{-6}$	$9.6 \times 10^{-6} \pm 2.4 \times 10^{-6}$
**1.0 µM NU172 + 1.0 µM AD**	49.3 ± 2.0	61.6 ± 3.0	$5.3 \times 10^{-3} \pm 1.4 \times 10^{-3}$	$6.1 \times 10^{-3} \pm 1.0 \times 10^{-3}$	$3.1 \times 10^{-5} \pm 1.4 \times 10^{-5}$	$6.1 \times 10^{-5} \pm 5.2 \times 10^{-6}$

[1.] clotting time (CT), viscous component (η′), elastic component (η″), the linear slope of the visocus or elastic component (m), thrombin aptamer (NU172), antidote (AD), nonsense antidote (NS_AD)

Supplementary Figures

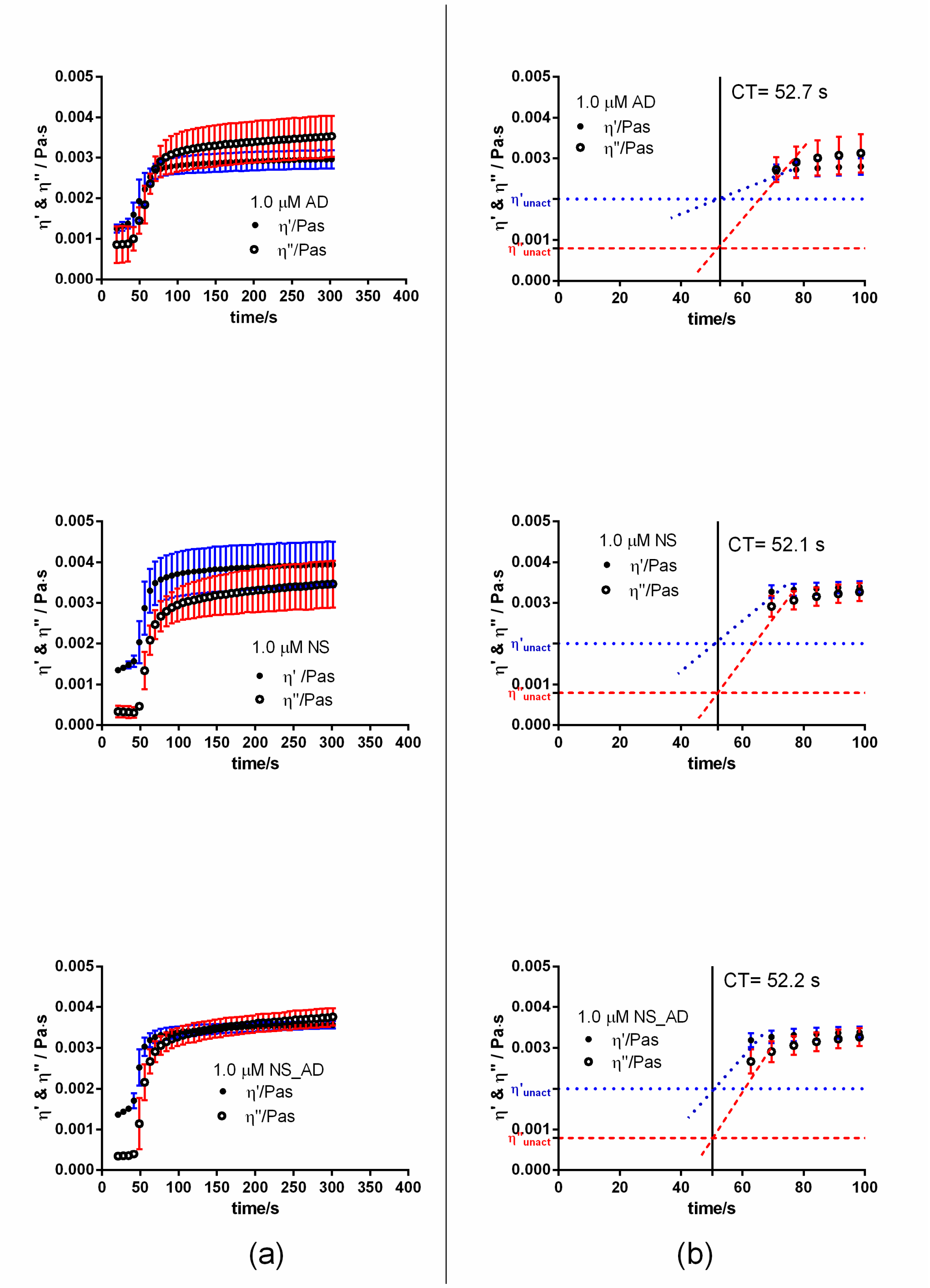

Figure S1. Detection of clotting time (CT) in citrated blood using PIEZ after the addition of 1.0 μM AD, NS, or NS_AD. (**a**) Detection of mean viscous (η′) and elastic (η″) components of blood at 100 Hz and 37 °C (n = 10 ± SD). (**b**) Enlarged parts of diagrams for the calculation of CT. The horizontal dotted blue line at 0.002 Pa·s visualizes the η′ and the dashed red line at 0.0008 Pa·s visualizes the η″ of unactivated citrated blood.

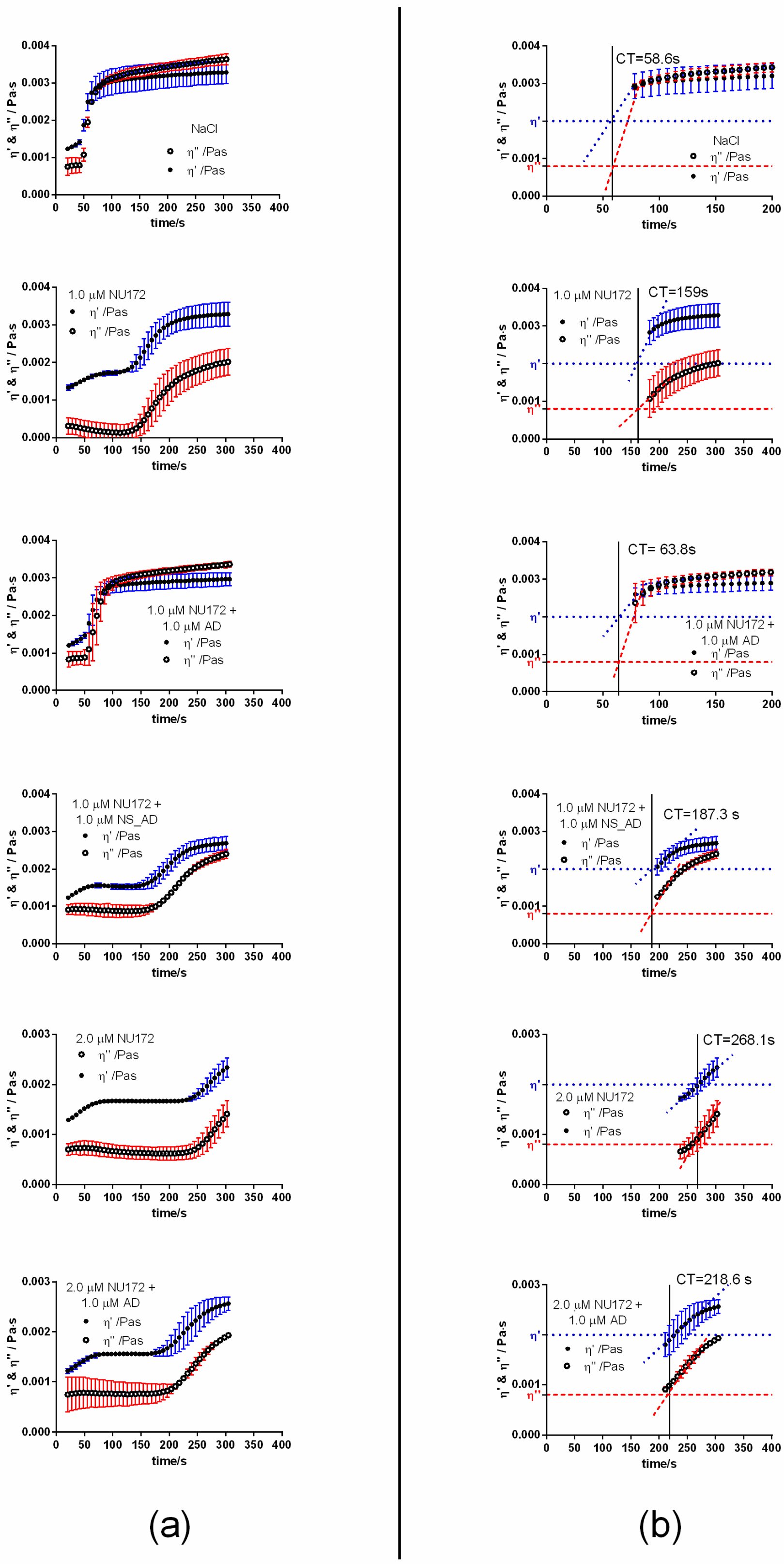

Figure S2. Detection of clotting time (CT) in citrated blood using PIEZ. After 2 min of incubation with 1.0 or 2.0 µM NU172, 1.0 µM AD or 1.0 µM NS_AD was added. Furthermore, CT was detected in blood containing NaCl, 1.0, or 2.0 µM NU172. (**a**) Detection of mean viscous (η′) and elastic (η″) components of blood at 100 Hz and 37 °C (n = 5 ± SD). (**b**) Enlarged parts of diagrams for the calculation of CT. The horizontal dotted blue line at 0.002 Pa·s visualizes the η′ and the dashed red line at 0.0008 Pa·s visualizes the η″ of unactivated citrated blood.
